# ACL Pacinian mechanoreceptors: Conceptualizing a vasoregulatory microtrauma healing function

**DOI:** 10.1002/jeo2.70305

**Published:** 2025-06-12

**Authors:** John Nyland, Jacob Williamson, Jonathon Lewis, Brandon Pyle, Jarod Richards, Ryan Krupp

**Affiliations:** ^1^ Norton Orthopedic Institute Louisville Kentucky USA; ^2^ Department of Orthopaedic Surgery University of Louisville Louisville Kentucky USA; ^3^ School of Medicine University of Louisville Louisville Kentucky USA

**Keywords:** anterior cruciate ligament, extracellular matrix, mechanotransduction, tissue healing, vasoregulation

## Abstract

**Purpose:**

Improved understanding of important natural anterior cruciate ligament (ACL) tissue functions may strengthen the case for their preservation. This commentary revisits human and comparative animal research studies of Pacinian mechanoreceptor histology, morphology and function, including those near the native proximal ACL and relates these findings to accumulated microtrauma healing and graft remodelling.

**Methods:**

Using the PubMed, Google Scholar and Web of Science databases, the medical literature was searched for peer‐reviewed information related to ACL morphology, knee joint circulation, ligament neuropeptides and vasoregulation, autonomic nervous system vasoregulation, Pacinian mechanoreceptors, ACL remodelling and nutrition, and psychological stressors and vasoregulation. Information from these searches was then synthesized into a conceptualization of a possible Pacinian mechanoreceptor role in primary ACL microtrauma healing and post‐surgical repair or reconstruction tissue remodelling.

**Results:**

In association with autonomic nervous system function, Pacinian mechanoreceptors in the ACL may serve an important vasoregulatory function in addition to proprioception.

**Conclusion:**

Pacinian mechanoreceptor‐mediated vasoregulation may be important for post‐sport performance or exercise training during primary ACL healing from accumulated microtrauma. Proximal remnant preservation during ACL reconstruction or repair may facilitate the angiogenic and neurogenic processes that drive native tissue and graft remodelling. Experimental and clinical studies are needed to confirm these concepts.

**Level of Evidence:**

Level V.

**Abbreviations**
3D: three‐dimensional
ACL: anterior cruciate ligament
ECM: extracellular matrix
IL: inferior lateral
ILGA: inferior lateral geniculate artery
IM: inferior medial
IMGA: inferior middle geniculate artery
MCL: medial collateral ligament
MGA: middle geniculate artery
NA: noradrenaline
NPY: neuropeptide‐Y
PCL: posterior cruciate ligament
REM: rapid‐eye movement
SL: superior lateral
SLGA: superior lateral geniculate artery
SM: superior medial
SMGA: superior middle geniculate artery
SNARE: soluble N‐ethylmaleimide‐sensitive factor activating protein receptor
VIP: vasoactive intestinal polypeptide

## INTRODUCTION

In addition to its essential mechanical functions [1], the anterior cruciate ligament (ACL) provides sensory information to the spinal cord, which prompts reflex ischiocrural neuromuscular tone and activation adjustments via gamma motoneurons [46, 51, 71]. This process increases dynamic knee joint stability [35, 102] and with effective training and cognitive engagement feed‐forward process development can further enhance injury prevention [20, 114]. Insufficiency of this ACL function, however, in association with meniscus injury, can lead to irreversible osteoarthritis [22, 53].

To better understand ACL injury aetiology and prevention, one needs to develop an intimate understanding of its risk factors and how to reduce them [24, 56, 61, 64]. Epidemiological, laboratory and clinical evidence of ACL mechanical fatigue‐related failure from accumulated microtrauma is growing [16, 40, 58, 73, 81]. Sudden rupture from non‐contact injuries may represent the culmination of a more longstanding condition [72, 73]. Since complete neuromuscular control restoration of the ACL reconstructed limb is rare and patients remain at risk for post‐traumatic osteoarthritis, improvements are needed [26, 28]. Improved understanding of important native knee joint tissue functions may strengthen the case for better preserving them during surgery. One such tissue is the proximal remnant of the torn ACL, which has a substantial mechanoreceptor density [7, 14, 35].

This commentary revisits human and comparative animal research studies of Pacinian mechanoreceptor histology, morphology and function, including those near the native proximal ACL and relates these findings to accumulated microtrauma healing. Using the PubMed, Google Scholar and Web of Science databases, the medical literature was searched for peer‐reviewed information related to ACL morphology, knee joint circulation, ligament neuropeptides and vasoregulation, autonomic nervous system vasoregulation, Pacinian mechanoreceptors, ACL remodelling and nutrition, and psychological stressors and vasoregulation. No year of publication or language restrictions were applied. Following title and abstract or synopsis review by the primary author (JN) and two co‐authors (JR, JW), information from selected studies was synthesized into a conceptual summary for how Pacinian mechanoreceptor‐mediated vasoregulation may influence primary ACL healing from accumulated microtrauma, surgical repair and graft remodelling. The following subsections were created: ACL Morphology, ACL Metabolism, Knee Joint Circulation, Ligament Neuropeptides and Vasoregulation, Autonomic Nervous System Vasoregulatory Control, Pacinian Mechanoreceptors, ACL Remodelling and Nutrition, Vasoregulation and Psychological Stressors, Summary, Framework for Future Experimental and Clinical Studies and Conclusion.

## ACL MORPHOLOGY

Cells within the ACL not only synthesize and secrete a diverse extracellular matrix (ECM) but also organize these constituents to meet three‐dimensional (3D) structure, stiffness and strength demands [47]. A major differentiation of the ECM is the basal lamina, a thin sheet‐like network composed primarily of laminin and Type IV collagen, which provides structural support and influences cell behaviour by guiding migration [110]. As it is positioned at the intersection between the femur and the tibia, the ACL is frequently exposed to 3D rotational loads throughout the lower extremity kinetic chain [38]. Approximately 90% of the ACL consists of parallel Type I collagen fibres. In addition, there are some glycosaminoglycans and elastin, and notably low fibroblast and fibrocyte cellularity. The ACL is primarily nourished by peripheral blood vessels from its superficial synovial sheath [79]. While the collateral ligaments can be perfused through various anastomotic circulatory connections, the cruciate ligaments have fewer options, primarily receiving vascularization from geniculate artery branches.

The total ACL cross‐sectional area is 18.42 ± 0.59, 45.64 ± 1.82 and 52.22 ± 1.32 mm^2^ at the femoral, middle and tibial levels, respectively [88]. Femoral cross‐sectional area is significantly smaller compared to the middle or tibial levels [88]. Longitudinal collagen fibre cross‐sectional area is 10.72 ± 0.45, 36.04 ± 1.6 and 41.77 ± 1.03 mm^2^ at the femoral, the middle and tibial levels, respectively [88]. Once again, femoral cross‐sectional area is less than that of the other levels. Synovium cross‐sectional area is 7.7 ± 0.24, 9.61 ± 0.72 and 10.46 ± 0.33 mm^2^ at the femoral, middle and tibial levels, respectively [88]. In summary, the femoral ACL attachment area has a smaller longitudinal collagen fibre and synovium cross‐sectional area than the middle or tibial insertion areas, placing it at greater accumulated microtrauma injury risk.

## ACL METABOLISM

Historically, ligaments were considered mechanical bands that did not respond to exercise [21]. In reality, the ligaments of a child or adolescent grow somewhat like the rings in a tree, primarily adding or removing collagen in adaptation to their loading state only at the ECM periphery [6]. Acute exercise training or sport performance bouts increase the need for collagen deposition; however, how ligament cellular processes respond to differing exercise training load frequencies, intensities and durations are largely unknown [45]. Ligament metabolic activity is low compared to muscles, bone and even cartilage [62, 98]. This unique characteristic allows ligaments to be more tolerant of, and resistant to the high forces to which they are repetitively subjected during running, jumping, sudden directional changes, and sports activities. The low metabolic rate and well‐developed anaerobic capacity of ligaments are essential to their ability to withstand prolonged continuous tension, reducing the risk of ischaemia and subsequent necrosis [38]. The diminished blood circulation that the ACL experiences during intense exercise in the presence of heightened efferent vasomotor nerve activation [31] enhances its biomechanical properties [73]. Lower metabolic needs account for reduced ligament internal vascularity [92]. However, naturally transient ligament blood flow influences essential amino acids nutrient availability before, during, and after exercise [99]. In this anerobic state, however, the ACL does not receive substantive healing nutrient inflow and metabolic waste product outflow. Survival in this state is enabled by the ligament's ability to recover during rest and lower intensity, less intense, more aerobic training activities [33]. The healing capacity of the ACL correlates with its degree of increased vascularity post‐injury [10].

## KNEE JOINT CIRCULATION

Ligament oxygen consumption is approximately 7.5 times less than that of skeletal muscle [98, 107]. The capacity for ligaments to withstand prolonged loads and sustain long periods of tension, while avoiding ischaemia and subsequent necrosis also makes them likely to heal more slowly post‐injury [113]. Healing effectiveness is directly related to the efficacy of ECM collagen synthesis and cross‐link development [22]. Poorly developed ECM leads to poorly organized ligament tissue with weakened biomechanical strength and increased injury risk [22].

**Figure 1:**
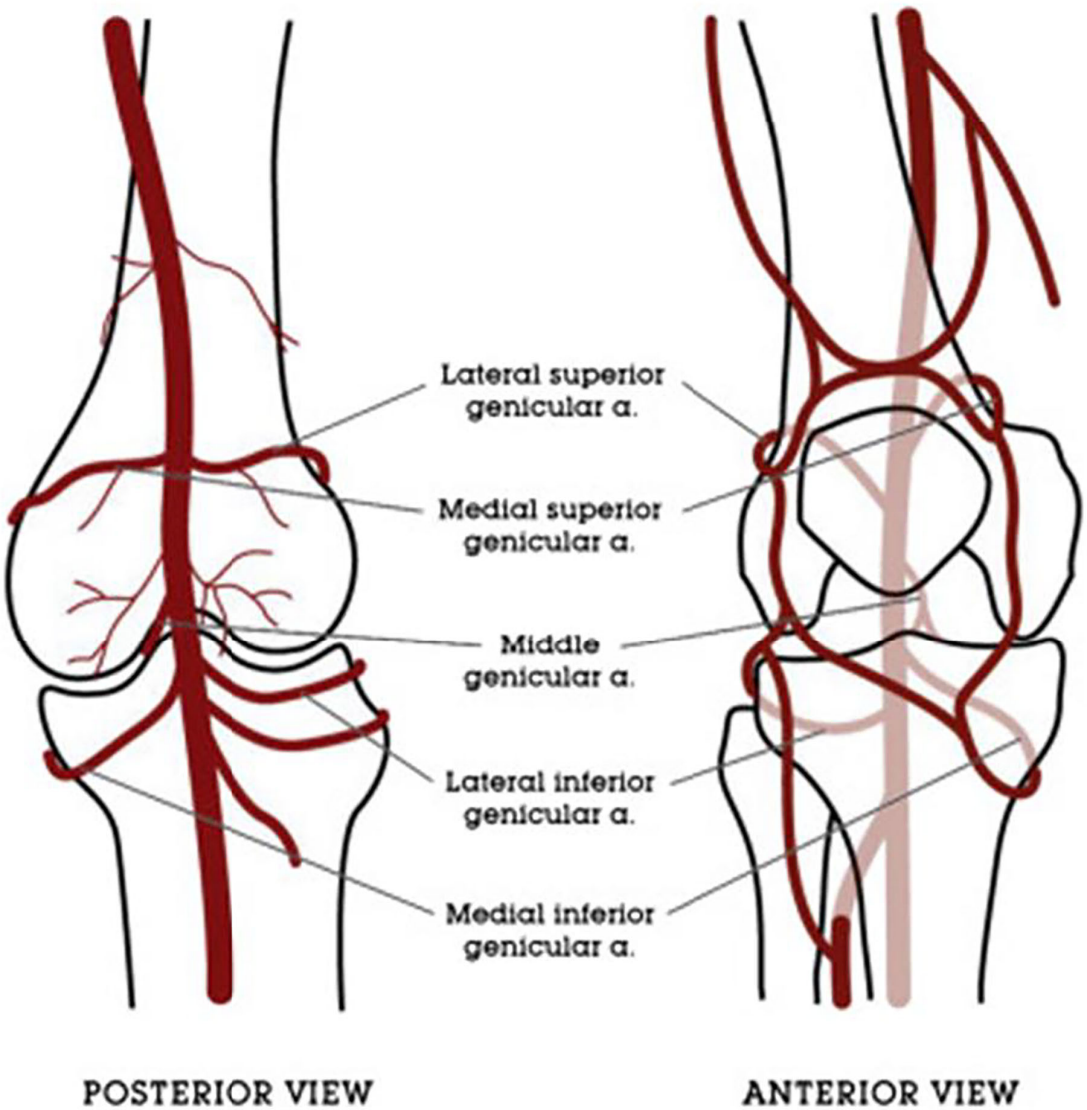
Geniculate artery collateral circulation [23]. Reprinted with permission of Elsevier Publishers.

**Figure 2:**
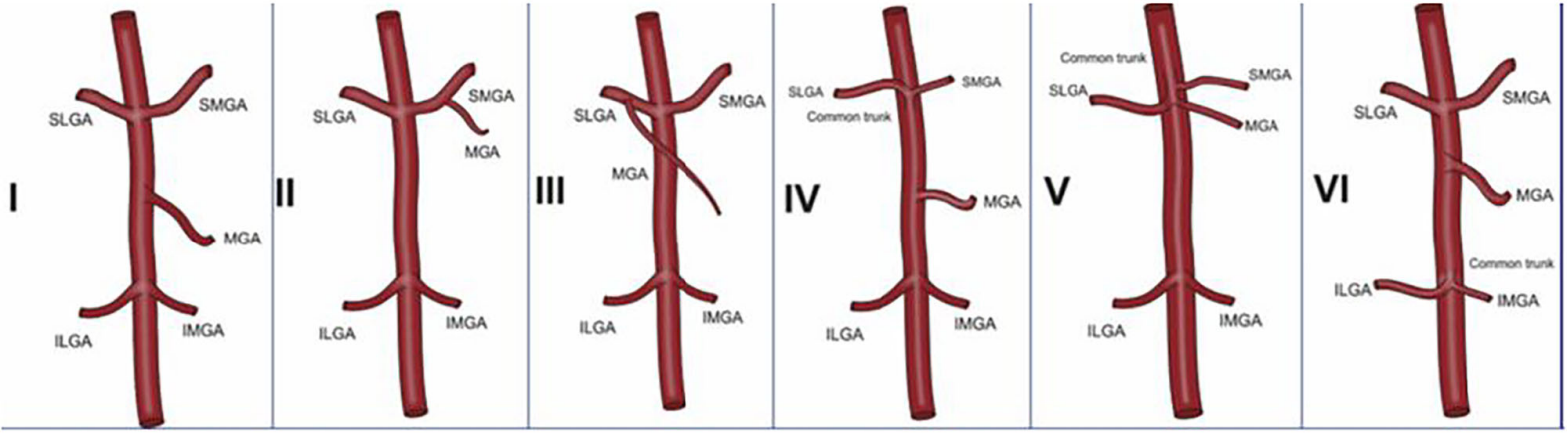
The six genicular artery branching sub‐types reported by Sighary et al. [101]. Type I (28%) represents an independent branching of the genicular vessels. The middle geniculate artery (MGA) branches off the superior middle geniculate artery (SMGA) in Type II (22%) and branches off the superior lateral geniculate artery (SLGA) in Type III (15%). There is a common trunk for the SLGA and SMGA in Type IV (15%), whereas Type V (10%) includes a common trunk for the SLGA, SMGA, and MGA. Type VI involves a common trunk for the inferior middle geniculate artery (IMGA) and the inferior lateral geniculate artery (ILGA) (6%). Reprinted with permission of Elsevier Publishers.

**Figure 3:**
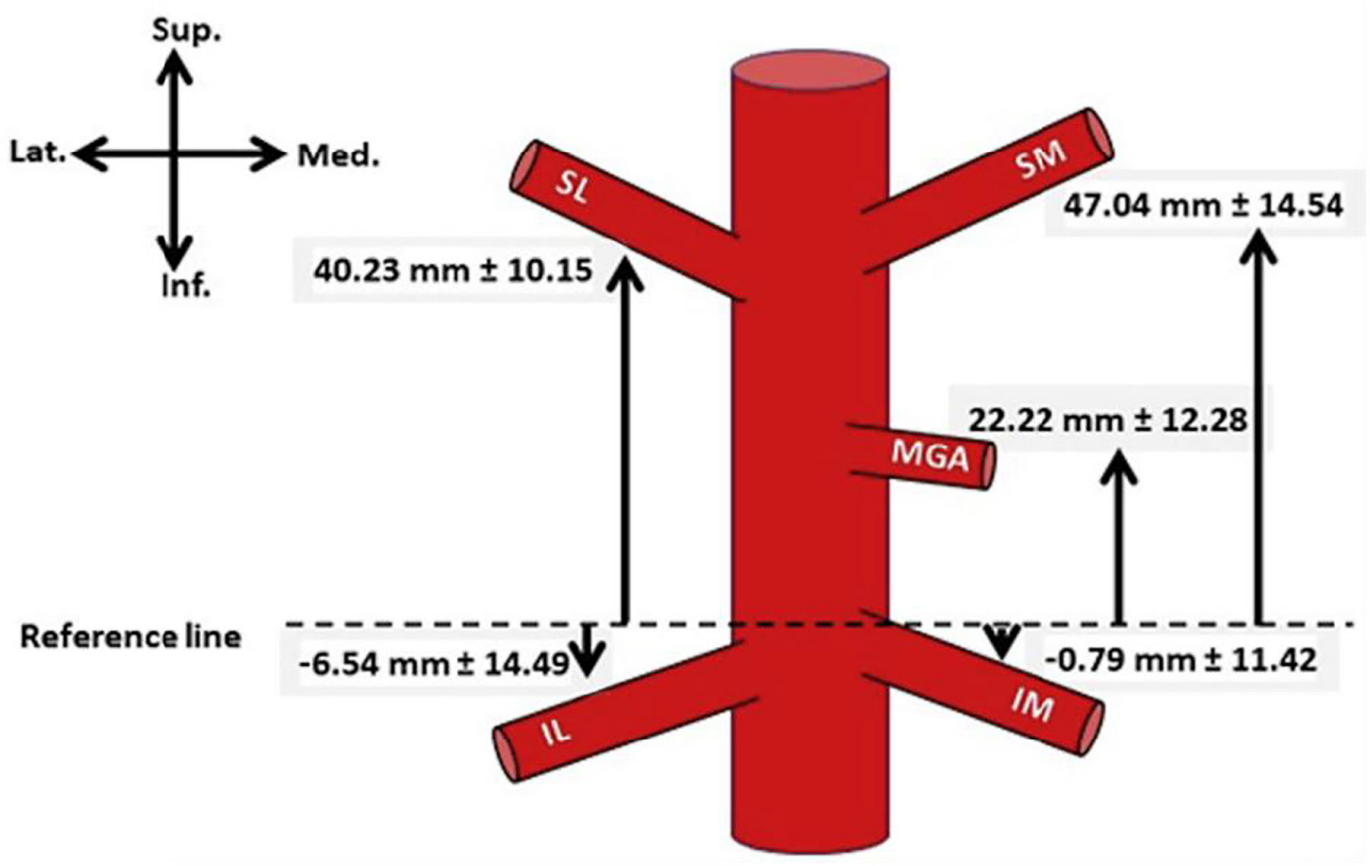
Genicular branch locations off the popliteal artery (mean ± standard deviation) [101]. Negative values represent the origin of the vessel below the reference line. IL, inferior lateral; IM, inferior medial; MGA, middle genicular artery; SL, superior lateral; SM, superior medial. Reprinted with permission of Elsevier Publishers.

The main blood supply to the lower limb and knee is provided by the femoral artery, subsequently becoming the popliteal artery after traversing the adductor hiatus [8]. Distal to the popliteal fossa, the popliteal artery branches into the anterior and posterior tibial arteries [8]. The knee joint vascular supply originates from three main vessels: The descending genicular artery traverses through the adductor hiatus before branching into three terminal branches (muscular, articular and saphenous). This supplies the superior knee region, while the anterior tibial recurrent artery, a branch of the anterior tibial artery, supplies the inferior knee region [8, 101]. The remainder of the knee joint receives a rich anastomosis from five genicular arteries originating from the popliteal artery, the superior medial and lateral, the middle (posterior) and the inferior middle and lateral genicular arteries (Figure 1) [6, 23, 101]. The medial knee is supplied by the superior medial genicular artery, middle genicular artery and inferior medial genicular artery; whereas the lateral knee is supplied by the superior lateral genicular artery and the inferior lateral genicular artery [23, 101]. In a study of 212 cadaveric knees, Sighary et al. [101] reported a high frequency of genicular vessel variation, with one of six general sub‐types existing in 72% of knees (Figure 2) and 4% not fitting any classification. From this information, they identified average genicular vessel location variations (Figure 3) [101].

The ACL gets its primary blood supply from the middle and inferior geniculate arteries, which provide branches to its synovial sheath [88]. The femoral ACL attachment has a dense, abundant blood capillary distribution [88] which penetrates it centripetally anastomosing with longitudinally oriented intraligamentous vessels [79]. Cross‐sectional blood capillary areas at the femoral, middle and tibial ACL regions are 1.24 ± 0.07, 0.35 ± 0.01 and 0.78 ± 0.06 mm^2^, respectively, with both the femoral and tibial regions possessing greater blood capillary area than the middle region [88]. Although ACL cross‐sectional area is smallest in the femoral insertion, the blood capillary area in this region is significantly greater than in the middle and tibial regions. Additionally, the number of sectioned capillaries is greatest in this area (327.9 ± 17.75), with both the femoral insertion region and tibial insertion region (204.18 ± 10.52) displaying significantly greater microcirculation density compared to the middle ACL region (104.95 ± 4.76) [88]. Based upon femoral ACL remnant capillary [88] and neural [35] density, conceivably, preservation during ACL reconstruction or repair may enhance the vasogenic and neurogenic responses that facilitate healing and remodelling.

## LIGAMENT NEUROPEPTIDES AND VASOREGULATION

Neuropeptide‐Y (NPY) serves a diverse role in neurotransmission, immune response, angiogenesis, and vasomotor regulation [68, 119]. Additionally, NPY may be an integrating factor between the sympathetic nervous system, blood vessels, and inflammatory responses to injury or stress [68, 119]. This potent vasoconstrictor contributes to peripheral vasomotor control in regulating post‐injury inflammatory responses [68]. Increased knee instability following ACL rupture is characterized by hyperemia, angiogenesis, inflammation and degraded articular tissue mechanical properties [68]. Prolonged increased sympathetic nervous system activity post‐injury may increase NPY tolerance with a downregulation of receptor expression [111]. Loss of this response eliminates one mechanism of moderating knee joint capillary bed hydrostatic pressure contributing to the effusion that alters tissue mechanical properties [68].

Following medial collateral ligament (MCL) rupture, the healing phases generally follow the order of haemorrhage, inflammation, proliferation, and remodelling [29, 63]. Unfortunately, partial ACL injuries possess limited healing potential and complete rupture generally exhibits no healing response [44]. Rupture of the ACL and osteoarthritis development also alter the normal homoeostatic mechanisms of other knee structures, including the MCL [67]. Using high‐resolution perfusion imaging after ACL transection, Miller et al. [67] reported that at 6 weeks post‐ACL deficiency, MCL blood flow increased 2.5 times.

Longstanding ACL deficiency results in long‐term degenerative joint changes and functional impairments as a result of both biologic and mechanical adaptations [66]. Following ACL transection, a strong angiogenic response occurs in the MCL including a loss of vasodilatory responses mediated through the endothelium and a loss of vasocontractile responses mediated through smooth muscle [66]. This leads to reduced collagen fibril organization, increased ligament thickness, ligament degeneration, and reduced mechanical strength [66]. Myofibroblasts have been identified in the intra‐articular remnant of ACL transected knees likely representing attempts by the injured tissue to stabilize the knee joint in the presence of chronically altered loading [66].

Compared to healthy control knees, in ACL transected knees, the MCL vasculature displays diminished responses to vasoactivity mediators phenylephrine, acetylcholine and sodium nitroprusside [66], with significantly increased vascular volume without significant angiogenic vessel smooth muscle and pericyte content changes [66]. Deficient vascular responsiveness is likely due to altered cellular signalling, impaired nitric oxide production and enhanced nitric oxide degradation related to the inducible nitric oxide synthase activity and inflammation observed in a degenerative chronically inflamed knee [66].

Posterior articular nerve branches from the tibial nerve containing both myelinated (2–10 µm diameter) and unmyelinated (about 1 µm diameter) axons travel close to the small blood vessels that innervate both the ACL and posterior cruciate ligament (PCL) [42]. The total axon number is >100 axons/cruciate ligament with about half of the total number located in the proximal third of both ligaments [42]. As with circulatory density, the proximal ACL is the richest section. Maintaining the integrity of the relatively hypoxic ACL is highly dependent upon its vasoregulatory status; however, we currently have a poor understanding of the mediators that regulate its blood flow [2].

The ACL blood vessels are innervated by three major nerve fibre types: sympathetic vasoconstrictors, parasympathetic vasodilators, and small diameter sensory vasodilators [2]. In rat knee collateral ligaments and joint capsules, Ackermann et al. [2] identified autonomic nerve fibres that contained NPY, noradrenaline (NA) and vasoactive intestinal polypeptide (VIP), which are known bone and joint blood flow regulators. NPY and NA were expressed in ligament and joint capsule tissues. While NPY potentiates the vasoconstrictive actions of NA, the co‐existence of VIP with NA or NPY likely represents vasoconstriction inhibition [49]. There were considerable differences, however, in the NPY and VIP levels in ligaments compared to joint capsules and tendons. While NPY and NA exert potent vasoconstrictive effects, VIP has vasodilatory effects [60]. The presence of vasoactive neuropeptides NPY, NA and VIP in perivascular ligament and joint capsule nerve fibres is the neuroanatomical basis for their vasoregulation. The main connective tissue blood flow regulators are NA and NPY, whereas VIP is more responsible for blood flow fine‐tuning at a more peripheral level [36, 68]. The coexistence of autonomic neurotransmitters in ligaments reflects both synergistic and inhibitory vasoactive effects. Ackermann et al. [2] reported that NPY concentrations were 15 times greater than VIP concentrations in ligaments, 2.5 times greater in tendons and 2.25 times greater in joint capsules. This suggests that ligament sympathetic tonus differs considerably from that in tendons or joint capsules [2]. The vasoactive neuropeptide concentration differences between these tissues likely influence blood flow, tissue healing, and degeneration susceptibility. Abnormal blood flow can diminish synovial fluid production and decrease ligament tensile strength from ischaemia and hypoxic degeneration [50]. Increased vasoactive NPY and NA levels have also been reported during stress and overtraining [57]. Conceivably, the combination of inherently poor ACL vascularization, increased and prolonged periods of vasoconstriction, and repetitive, high‐frequency or intensity mechanical loads during exercise training or sport performance may promulgate the ischaemia and hypoxic degeneration that precedes sudden failure from non‐contact mechanical fatigue‐related injury mechanisms [2]. Vasoactivity dysregulation involving autonomic neuropeptides may be an underlying mechanism in ligament inflammatory and degenerative disorder development [2].

## AUTONOMIC NERVOUS SYSTEM VASOREGULATORY CONTROL

The autonomic nervous system is critical to mediating the cardiovascular adjustments that serve the metabolic demands of exercising muscles [27]. Additionally, sympathetically mediated vasoconstriction in non‐exercising muscles and visceral organs (e.g., splanchnic circulation) facilitates cardiac output redistribution to active skeletal muscles. Associated with this, normal sympathetic nervous system vasoconstriction is attenuated in active muscles in part due to an effect of muscle metabolites that diminish vasoconstrictor responses to gamma adrenergic receptor activation [27, 48]. This modulation or ‘functional sympatholysis’ is a protective mechanism that optimizes working muscle blood flow during the increased sympathetic vasoconstrictor drive that occurs during exercise training or sport performance [27, 48].

Autonomic nervous system knee joint innervation is involved in stretch or pain sensation reception, immune system responses, and trophic actions [48, 55, 97]. Using a rat model, Schwab et al. [97] identified rich artery and arteriole innervation with calcitonin gene‐related peptide and neurokinin A producing nerve fibres, especially along fibrous crucial ligaments, concentrated in the insertion regions and in subsynovial tissue suggesting effective microcirculation regulation [97]. The neural reflex mechanisms that reinforce parasympathetic and sympathetic nervous system adjustments during exercise training or sport performance involve central command, the exercise pressor reflex, the arterial baroreflex, and cardiopulmonary baroreceptors, with further modulation from arterial chemoreceptors and phrenic afferents from respiratory muscles (i.e., respiratory metabolic reflex) [27]. Each of these mechanisms modulates autonomic nervous system adjustments during exercise or sport performance, interactively orchestrating the needed cardiovascular response in an intensity‐dependent manner [27].

Increased heart rate during low‐intensity, steady‐state dynamic exercise is primarily driven by reduced cardiac parasympathetic nervous system activity, whereas the relative contribution of sympathetic mechanisms occurs as exercise training intensity increases [27]. Exercise duration and intensity both influence how the sympathetic nervous system responds to dynamic exercise. Heart rate increases approximately linearly with oxygen uptake during incremental dynamic exercise [27]. A normally functioning autonomic nervous system is essential to having an appropriate cardiac response during exercise, which has important exercise training and sport performance implications [27].

## PACINIAN MECHANORECEPTORS

**Figure 4:**
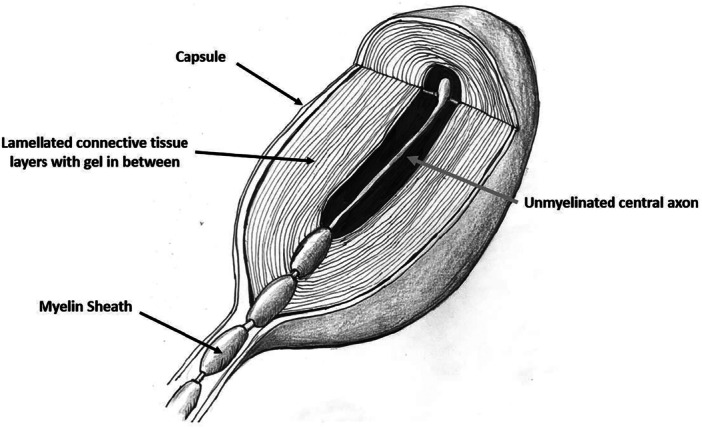
Pacinian corpuscles are rapidly adapting, low‐threshold mechanoreceptors that are responsive to vibration or deep pressure. They are activated at the onset or cessation of joint movement when sudden tissue stress changes occur. Each has a single myelinated axon which loses its sheath near the corpuscle centre. The capsule consists of 20–70 concentrically arranged connective tissue lamellae around the central axon terminal forming an ‘onion‐appearing’ structure. The capsule consists of fibroblasts and fibrous connective tissue (mainly Type IV and Type II collagen), separated by gelatinous material (92% water). As the lamellae slide past each other during deformation from external pressure, it selectively responds to low‐frequency stimuli, dissipating the effects of sustained external pressure, acting as a physiological high‐pass filter (Wikipedia https://en.wikipedia.org/wiki/Pacinian_corpuscle).

Zimny et al. [118] identified two morphologically distinct mechanoreceptors in the human ACL (Ruffini and Pacinian). The total number of mechanoreceptors in the cruciate ligaments is greater than in other ligaments or tendons of the knee, highlighting their crucial role in knee joint proprioception [14]. Early reports of Pacinian mechanoreceptors in the knee collateral and cruciate ligaments described them as being particularly sensitive to high velocity tissue length changes serving an important proprioceptive position sense function [51]. Pacinian or Type II mechanoreceptors are dynamic, adapt rapidly and possess a low activation threshold [7]. They are inactive in immobile joints, becoming active only at the onset or cessation of joint accelerations at which sudden tissue stress changes occur. They are essential motion sensors, not position sensors [7]. In the abdominal viscera, pressure‐induced afferent impulses from Pacinian mechanoreceptors help mediate the reflex circulatory responses that regulate arterial pressure [91]. Conceivably, those located within the ACL may function similarly. The unique design of these lamellar‐structured receptors makes them particularly sensitive to direct pressure, vibration or pulsatile stimulation (Figure 4). Pacinian mechanoreceptors are widely distributed throughout the mesentery, serous membranes, lymph nodes, and connective tissues [32, 53, 69, 78, 84, 89, 93, 103]. Pacini‐like lamellar mechanoreceptors have also been identified in the brachial, radial, ulnar, femoral, popliteal, post‐tibial and peroneal artery adventitia [74, 85, 112, 116].

In the presence of neuropeptide NA Pacinian mechanoreceptors modulate sympathetic nervous system tonus responses to mechanical deformation or displacement [94] and sympathetic postganglionic nerve fibres that enter its core provide a mechanosensitive gating effect [94]. These mechanoreceptors were first observed by Vater [109], who reported that they were attached in large numbers to digital nerves. Almost 100 years later, Pacini [75] re‐discovered them describing their lamellar structure and giving them his name. Glees et al. [37] observed that in cat mesentery, blood vessels entered Pacinian mechanoreceptors at the same location as the nerve axon supplying the central core with capillary loops. Arndt [4] reported that Pacinian mechanoreceptors were associated with blood vessel function. Thoma [105] identified a considerable number of Pacinian mechanoreceptors within the aortic system, suggesting that they were involved in blood pressure regulation, while Grosser [41] observed a series of Pacinian mechanoreceptors near the arterio‐venous anastomoses in the limbs of a bat suggesting that this indicated anastomosis activity. Michailow [65] reported that the extensive Pacinian corpuscle distribution in subcutaneous tissue, muscles, near joints, periosteum, in viscera, and in blood vessel adventitia, suggested that they were responsible for peripheral circulatory pressure regulation. Schumacher [96] found groups of Pacinian mechanoreceptors located near the human glomus coccygeum which he described as a collection of arterio‐venous anastomoses, suggesting that they might be a ‘moisture regulator’, taking up moisture when tissue fluid increased from enlarged capillaries and, as a result, the nerves of the inner bulb would be compressed causing the arterio‐venous anastomoses to open capillary circulation so that tissue fluid was reduced. Schumacher [95] later expanded his previous theory suggesting that the adventitial Pacinian mechanoreceptors of blood vessels were stimulated by arterial pulsation pressure, reporting that feline Pacinian mechanoreceptors expanded when mesentery arterial pressure increased. In also studying Pacinian mechanoreceptors in feline mesentery blood vessels, Sheehan et al. [100] suggested that their impulses might cause reflex blood vessel contraction and dilatation through sympathetic nerve activation. Woollard and Weddell [116] considered that ‘lamellar corpuscles’ in limb blood vessel adventitia might be a vascular reflex starting point and that the afferent reflex component was activated by vascular blood pressure changes. In studying Pacinian mechanoreceptor impulse discharge from feline mesentery, Gammon and Bronk [34] suggested that elevated intravascular pressure signalled blood vessel distention. Gray and Malcolm [39] also studied mesenteric Pacinian mechanoreceptor nerve impulses suggesting that they originated from capillary circulation mechanical stimulus to the axon [15].

Pacinian mechanoreceptor blood vessels are derived from larger vessels proximal to the arterio‐venous anastomoses, therefore anastomosis activity cannot directly influence their blood supply [15]. Their veins, however, join anastomosis outlets and therefore Pacinian mechanoreceptor drainage is dependent upon the state of anastomosis activity. When the anastomosis is closed, blood from Pacinian mechanoreceptors can flow freely to the venous network, but when the anastomosis opens and the arterial blood fills its outlets, the pressure may stop or even tend to reverse Pacinian mechanoreceptor vein flow [15]. Since the Pacinian mechanoreceptor cannot expand, increased outer bulb pressure is transmitted to receptors within its inner bulb. In this way, the Pacinian mechanoreceptor structure and blood vessel pattern may serve as a mechanism that signals local blood supply changes in association with arterio‐venous anastomosis activity [15]. The extensive non‐nervous outer bulb and presence of blood vessels within it suggest that it may modulate tissue capillary circulation [15].

The mechanoreceptors first observed by Vater [109] and described by Pacini [75] were perceived by Thoma [105] to be involved with vascular tone regulation. Schumacher [95] observed that their shape changed with mesenteric vascular blood pressure changes. Around this time, Rainer [82], Van de Velde Vox [108] and later Collin [17] viewed their function as being associated with vascular tone regulation. Some Pacinian mechanoreceptors are intimately related to the digital arteries from which they derive their blood supply and in these instances, their veins also serve as joint arteriovenous anastomosis outlets [15]. Compared to cats and dogs, in humans, Pacinian mechanoreceptor distribution is greater in the adventitia of the posterior tibial and anterior tibial blood vessels than in the femoral vessels [85].

Feline knee joint Pacinian mechanoreceptors activate in synchrony with the arterial pulse [12, 34, 106, 117]. As the knee flexes, an artery in the limb gets bent and compressed [54] causing turbulent blood flow within the bent vessel [61, 115]. Although their precise function remains unknown [9], Pacinian mechanoreceptors provide a negative feedback control system function to reduce arterial pressure fluctuations from stressful stimuli, sympathetic nervous system activation, and increased heart rate during sport performance or exercise training [18, 19]. Some have proposed that they serve a ‘self‐shunting’ vasoregulatory function as a variable conductance transducer converting mechanical pressure sensations into neural activation [9, 59].

There is anatomical, physiological and pharmacological evidence suggesting that human Pacinian mechanoreceptor sensitivity is modified by sympathetic nervous system activation [43, 90, 104]. Increased sympathetic nervous system activation during stress is often accompanied by momentarily increased blood pressure and/or pulse pressure wave amplitude changes that are transmitted to the tissues surrounding small blood vessels prompting Pacinian mechanoreceptor excitation [30]. Pacinian mechanoreceptors are extremely sensitive to rapid acceleration, even when it occurs some distance away from their physical location [43].

At exercise onset, arterial pressure, heart rate and cardiac output increase [86] and sympathetic nervous system outflow also increases. During exercise, sympathetic nervous system activation regulates blood flow to working muscles in a physiologically advantageous manner [18, 19]. There are two primary baroreflex regulation factors during exercise: central command and afferent input from active skeletal muscles [87]. Central command refers to effects mediated by descending pathways from the motor cortex, while afferent input refers to input from skeletal muscle chemoreceptors activated by increased metabolic activity and mechanoreceptors activated by muscle contraction [18, 19]. Afferent input from muscle chemo‐ and mechanoreceptors causes a resetting of the vasomotor component of the baroreceptor reflex [83]. During periods of increased mental stress, the baroreflex control of sympathetic nervous system vasomotor activity operates over a greater arterial pressure range with increased gain [18, 19].

## ACL REMODELLING AND NUTRITION

Growing evidence suggests that non‐contact mechanical fatigue‐related ACL injuries may be associated with cumulative microtrauma [16, 40, 58, 73, 81]. Conceivably, this overuse impairs the capacity of the ACL ECM to naturally remodel in the presence of a homoeostasis imbalance. Lamellated mechanoreceptors have been identified between ACL and PCL collagen fibre bundles, mostly in their proximal regions and in the knee joint capsule tending to be located in close proximity to blood vessels [42], thus potentially serving a vasoregulatory function. In the presence of glutamate, glutamate receptors, glutamate transporters and SNARE proteins, synaptic‐like activity in Pacinian mechano‐receptors has been described within its inner‐core lamellae, at interlamellar connections, where the lamellae contact the axon membrane, and at the lamellar tips [73, 76, 110].

Circulatory system amino acid delivery to the ACL supports collagen synthesis [3]. After exercise training, increased growth hormone and insulin‐like growth factor production are the primary stimulators [3]. With its limited vascularity, optimizing amino acid delivery to meet the demands of high‐volume exercise training is essential [54]. To take advantage of peak blood flow timing, protein sources that quickly attain high circulating amino acid levels are considered optimal [3]. Consistent essential amino acid intake from supplemental and/or food protein sources delivered at key periods (i.e. prior to or following exercise) may improve ACL tissue health [3].

Using an engineered ligament model, and an intermittent exercise programme (10 min of activity, 6 h of rest), Paxton et al. [77] reported that at 5 days post‐exercise engineered ligaments that had undergone an intermittent activity protocol produced more collagen than those that were exercised continuously. These findings agree with what occurs to bone in vivo, with a limited number of loading events followed by 6–8 h of rest creating greater bone mineral deposition [13]. In summary, ligaments get maximal collagen synthesis stimulus from short activity periods with comparatively longer rest [99]. Nutrient supplementation (gelatin and vitamin C) post‐exercise augments collagen synthesis with an accelerated rate occurring as early as 4 h post‐exercise, and 5 h following pre‐exercise nutrient supplementation, being maintained for 73 h [99].

Pacinian mechanoreceptors are present in ACL remnant tissue; however, their number may decrease with time post‐injury [35, 52]. With the onset of intra‐articular arthroscopic ACL reconstruction and greater appreciation for ‘anatomical’ graft placement, to improve placement accuracy complete proximal ACL remnant debridement is often performed. Removal of this vital tissue, however, may decrease graft neurogenic and angiogenic recovery potential during the remodelling and ligamentization process [70, 88].

## VASOREGULATION AND PSYCHOLOGICAL STRESSORS

Homoeostasis assumes that physiological functions are controlled around a set‐point and that system changes occur from feedback regulation to re‐establish ‘steady‐state’ consistency [5]. Both plasma cortisol and growth hormone levels peak several times during sleep and are strongly influenced by habitual physical activity levels and sleep characteristics [5]. Circadian blood pressure and heart rate rhythms are highly influenced by sleep, activity, posture and nutrition [5].

Sympathetic nervous system vasoregulation changes according to an individual's behavioural state [19]. During both rapid‐eye movement (REM) and non‐REM sleep, rat sympathetic nervous system activity is decreased [19]. During exercise or mental stress, increased arterial pressure increases blood flow to working skeletal muscles and to the heart in response to actual or potentially increased metabolic demands [19]. In contrast, during sleep, arterial pressure decreases with reduced cardiac work while maintaining blood flow perfusion to vital organs [19], conceivably including the ACL [72].

Growing evidence suggests that psychosocial factors influence the balance between physical or mental stress and recovery [11]. For example, athletes with reduced sleep duration or quality, those with stronger athletic identities, with greater perceived life stress, or with perfectionist traits are at greater overuse injury risk [11]. During rest and recovery, the parasympathetic nervous system facilitates vasodilation to internal organs and other tissues that receive minimal or no blood flow during exercise training or sport performance. This important process may also potentially drive ACL tissue recovery and remodelling from accumulated microtrauma [72, 73].

A variety of stressors can upregulate sympathetic nervous system activity. When these stressors are excessive or prolonged, sleep quality and duration may decrease. In addition to increased stress‐related plasma cortisol levels, decreased sleep quality and duration are associated with reduced tissue healing from accumulated microtrauma [72, 73]. Even compared to a few years ago, child and adolescent athletes have greater potential stressor exposure frequency, duration and total volume in the form of more specialized elite type sport performance or exercise training, increased computer screen time, increased cell phone and ear bud use, increased environmental noise, and other multi‐modal environmental pollutants [72]. The summative negative effects of these stressors on sleep quality and duration upregulate the sympathetic nervous system. These responses prolong vasoconstriction beyond exercise training or sport performance into what should be the vital parasympathetic nervous system, ECM recovery and remodelling time that athletes need between repetitive training sessions, practices, and competitions [73]. Within this context, greater appreciation of the possible role of Pacinian mechanoreceptors serving as ACL blood flow regulators should strengthen the case for their preservation during surgery to better facilitate ACL graft or repair healing and remodelling [25, 26]. Although the ACL is designed for anerobic metabolism, low velocity, non‐contact, deceleration injuries are common [38, 80], and perhaps more likely when regular active rest and recovery periods are insufficient to reverse the accumulated microtrauma that precedes sudden non‐contact mechanical fatigue‐related failure [40, 73].

## SUMMARY

An abundance of historical evidence supports the concept that Pacinian mechanoreceptors may serve an important vasoregulatory function. In the ACL, many of these mechanoreceptors exist in both the proximal and distal regions, which are more susceptible to non‐contact mechanical fatigue‐related failure from accumulated microtrauma. ACL metabolism, ECM healing and remodelling that should occur in synchrony with the intermittent loads associated with sport performance or exercise training may be largely mediated by autonomic nervous system vasoregulation. Greater appreciation for this possible function may influence recommendations regarding athlete sport performance or exercise training volume and recovery (primary injury prevention), and the clinical interpretation of diagnostic images of the proximal ACL during plan of care and surgical technique decision‐making regarding graft placement and remnant preservation (secondary prevention). Further study is needed to confirm these concepts.

## FRAMEWORK FOR FUTURE EXPERIMENTAL AND CLINICAL STUDIES

Previous human and comparative animal studies have supported the concept of Pacinian mechanoreceptors possessing a vasoregulatory function. Experimental studies are necessary to confirm this in the human ACL. If identified, clinical studies would then be indicated to confirm autonomic nervous system‐mediated vasoregulatory ACL blood supply control under different sport performance or exercise training loading and rest conditions. Following this, further studies would be indicated to confirm optimal nutrient characteristics and concentrations, delivery timing and how they might accelerate ACL ECM remodelling to enhance the biomechanical properties that might reduce the effects of accumulated microtrauma from the high volume sport performance or exercise training that often precedes non‐contact sudden mechanical fatigue‐related failure. Finally, clinical studies would be needed to confirm that adherence to a specific sport performance or exercise training and recovery plan would lead to reduced injury occurrences.

## CONCLUSION

Pacinian mechanoreceptor vasoregulatory function may be of potential importance for post‐sports performance or exercise training to better facilitate primary ACL healing from the effects of accumulated microtrauma. Proximal remnant preservation during ACL reconstruction or repair may also help facilitate the angiogenic and neurogenic processes that drive tissue remodelling and graft ligamentization. Experimental and clinical studies are needed to confirm these concepts.

## AUTHOR CONTRIBUTIONS

Conceptualization: John Nyland. Literature search and data analysis: John Nyland, Jacob Williamson, Jarod Richards. Drafted and critically revised the work: John Nyland, Jacob Williamson, Jonathon Lewis, Brandon Pyle, Jarod Richards and Ryan Krupp. All authors approved the final work.

## CONFLICT OF INTEREST STATEMENT

The authors declare no conflicts of interest.

## ETHICS STATEMENT

The ethics statement is not available.

## Data Availability

Data Availability Statement is not available.
